# Acetylcholine in action

**DOI:** 10.7554/eLife.57515

**Published:** 2020-05-19

**Authors:** Erin M Wall, Sarah C Woolley

**Affiliations:** 1 Integrated Program in Neuroscience, McGill UniversityMontrealCanada; 2 Center for Research on Brain, Language, and Music, McGill UniversityMontrealCanada; 3 Department of Biology, McGill UniversityMontrealCanada

**Keywords:** songbird, acetylcholine, HVC, motor performance, Other

## Abstract

The neurotransmitter acetylcholine influences how male finches perform courtship songs by acting on a region of the premotor cortex called HVC.

**Related research article** Jaffe PI, Brainard MS. 2020. Acetylcholine acts on songbird premotorcircuitry to invigorate vocal output. *eLife* **9**:e53288. doi: 10.7554/eLife.53288

Acetylcholine is a neurotransmitter that helps organisms filter the vast amounts of information received from the environment. In the sensory cortex, it acts by fine-tuning the activity of neurons to heighten attention, which helps with learning and memory (Sarter and Lustig, 2019; Lee and Dan, 2012; Picciotto et al., 2012).

Heightened attention also boosts the precision and speed of movements (Song, 2019). Previous research in this area has focused on neuromodulation in the basal ganglia, a group of neural structures in the forebrain that help to select, initiate, maintain, and adapt motor actions (Berke, 2018; Mink, 1996; Turner and Desmurget, 2010). For example, dopamine is an important neurotransmitter in this region, and its loss is associated with movement disorders such as Parkinson’s disease. Disrupting acetylcholine signaling also leads to problems with movement, yet the influence of acetylcholine on motor performance is not fully understood (Conner et al., 2010). Now, in eLife, Paul Jaffe and Michael Brainard from the University of California, San Francisco report the results of experiments on songbirds that shed light on the relationship between acetylcholine, arousal, and motor performance (Jaffe and Brainard, 2020).

The team took advantage of the fact that male Bengalese finches naturally alter their song performance depending on their audience. Each male has his own song that he rehearses alone. However, when aroused and courting a female, the male produces a song that is more stereotypical (less variable), longer, and faster (Figure 1A; Sakata et al., 2008). Several specialized neural circuits – some in the cortex, and some in the basal ganglia – are required to learn and produce songs. Researchers can monitor and manipulate these circuits with precision to understand their function (Sakata and Woolley, 2020).

**Figure 1.**
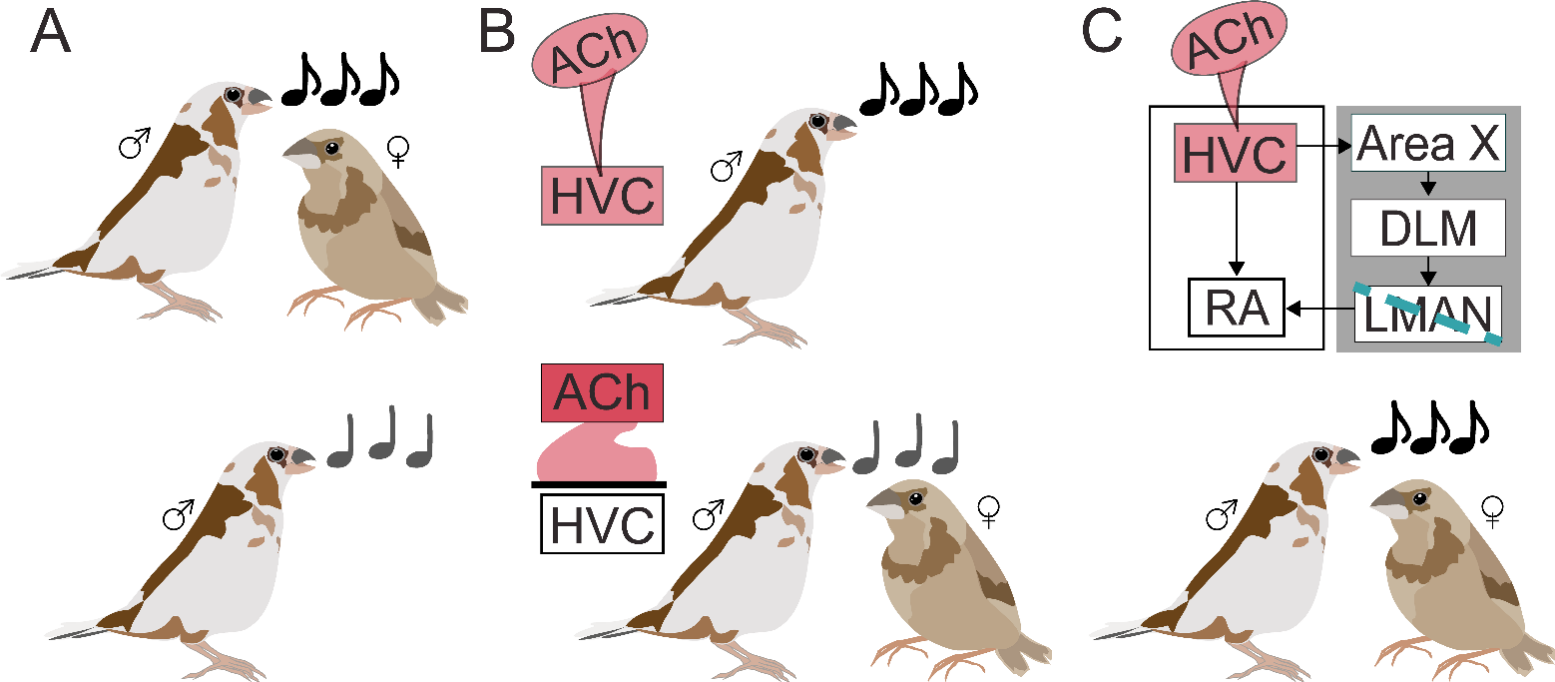
Acetylcholine invigorates motor performance. (**A**) During courtship (top), male Bengalese finches sing louder, faster, more stereotyped songs (black notes) to females compared to when they sing alone (grey notes; bottom). (**B**) Using a combination of local drug infusion and electrophysiological recordings, Jaffe and Brainard demonstrated that changes to song performance may depend on acetylcholine (Ach) acting in the premotor cortical nucleus HVC. When their HVC was stimulated with a drug mimicking acetylcholine (pink infusion; top panel), male finches produced songs similar to courtship songs, despite being alone. On the other hand, blocking acetylcholine naturally released in HVC during courtship singing (bottom panel) made the courtship song performance more similar to non-courtship song even when females were present. (**C**) HVC is connected to the robust nucleus of the arcopallium (RA) – a region involved in motor vocal output – both directly and through a cortical-basal ganglia circuit (gray box) that involves the basal ganglia nucleus (Area X), the dorsolateral anterior thalamic nucleus (DLM), and the lateral magnocellular nucleus of the anterior nidopallium (LMAN). Creating a lesion in LMAN (blue line) while stimulating HVC with acetylcholine preserved vocal vigor, showing that the neurotransmitter can act independently from the cortical-basal ganglia circuit.

First, Jaffe and Brainard focused on a premotor cortical region called HVC, where they locally infused a drug that mimics the effects of acetylcholine. As a result, males started to sing as if a female were present: songs were faster, louder, and less variable during drug infusion than in control conditions (Figure 1B). Neurons in HVC also started to show the same type of pattern observed during courtship singing towards females – there was, in particular, neural activity increased. Together, these experiments suggest that acetylcholine plays a role in shaping singing behavior in a social context.

Next, they assessed whether differences in behaviour in the presence and absence of a female normally depend on acetylcholine. To this end, Jaffe and Brainard blocked specific acetylcholine receptors, leading to courtship songs in the presence of females becoming lower in pitch, more variable, and altogether more similar to songs performed alone (Figure 1B). Decreasing acetylcholine activity in HVC therefore weakened the vigor of courtship singing, revealing that acetylcholine can drive changes in the brain that energize male performances towards females.

In the brains of songbirds, HVC is connected to the region that controls vocal motor outputs both directly and through a separate circuit that goes through the basal ganglia. Jaffe and Brainard therefore set out to determine which of these pathways acetylcholine acts on to enhance the vigor of the song. They disrupted the circuit that connects the basal ganglia to the vocal output region and showed that, in this context, increased acetylcholine activity in HVC still produced the same enhanced singing behavior (Figure 1C). This demonstrates that acetylcholine can invigorate song performance even without the basal ganglia being involved.

From songbirds to humans, many vertebrates rely on ‘prosodic cues’ such as pitch and tempo to convey motivations and emotions during communication (Pell et al., 2009; Sakata and Vehrencamp, 2012). Knowing how acetylcholine heightens motor performance sheds light on the neural circuits that underlie the production of these cues. Other chemicals, such as dopamine and norepinephrine, also fine-tune the activity of neurons in motor circuits. In the future, understanding how acetylcholine interacts with these neurotransmitters, both in overlapping and independent regions, will be necessary to fully grasp how arousal influences motor behavior.
